# Clustered Federated Spatio-Temporal Graph Attention Networks for Skeleton-Based Action Recognition

**DOI:** 10.3390/s25237277

**Published:** 2025-11-29

**Authors:** Tao Yu, Sandro Pinto, Tiago Gomes, Adriano Tavares, Hao Xu

**Affiliations:** 1 Centro Algoritmi, University do Minho, 4800-058 Guimarães, Portugal; id8670@alunos.uminho.pt (T.Y.); sandro.pinto@dei.uminho.pt (S.P.); mr.gomes@dei.uminho.pt (T.G.); atavares@dei.uminho.pt (A.T.); 2 College of Computer Science and Technology, Jilin University, Changchun 130012, China

**Keywords:** skeleton-based action recognition, spatio-temporal graph attention, clustered federated learning, inter-cluster regularization

## Abstract

Federated learning (FL) for skeleton-based action recognition remains underexplored, particularly under strong client heterogeneity where regular FedAvg tends to cause client drift and unstable convergence. We introduce Clustered Federated Spatio-Temporal Graph Attention Networks (CF-STGAT), a clustered FL framework that leverages attention-derived spatio-temporal statistics from local STGAT models to dynamically group clients and perform attention-weighted inter-cluster fusion that gently align cluster models. Concretely, the server periodically extracts multi-head parameter-based attention descriptors, normalizes and projects them via PCA, and applies K-means to form clusters; a global reference is then computed by attention–similarity weighting and used to regularize each cluster model with a lightweight fusion step. On NTU RGB+D 60/120(NTU 60/120), CF-STGAT consistently outperforms strong FL baselines with the STGAT backbone, yielding absolute top-1 gains of +0.84/+4.09 (NTU 60, X-Sub/X-Setup) and +7.98/+4.18 (NTU 120, X-Sub/X-Setup) over FedAvg, alongside smoother per-client trajectories and lower terminal test loss. Ablations indicate that attention-guided clustering and inter-cluster fusion are complementary: clustering reduces within-group variance whereas fusion limits cross-cluster divergence. The approach keeps local training unchanged and adds only server-side statistics and clustering.

## 1. Introduction

Skeleton-based human action recognition (HAR) is an active area in computer vision with applications in intelligent surveillance and robotic perception [1,2]. Given a sequence of 3D skeletal joints, the goal is to predict the action label by jointly modeling spatio-temporal configurations and dynamics. Early CNN/RNN approaches often treated skeletons as images or plain sequences and thus underutilized the explicit skeletal graph structure. Graph neural networks (GNNs) address this by operating on graph-structured data; the landmark ST-GCN [3] constructs a spatio-temporal skeleton graph and achieves strong centralized performance. Also, recent work [4] has demonstrated the effectiveness of spatial–temporal graph convolutional networks for skeleton-based activity recognition in practical applications. Subsequent advances enrich spatial topology modeling and temporal expressiveness through two-stream or channel-wise variants and multi-scale graph designs [5,6,7]. To capture long-range interactions across distant joints and timesteps, the Spatio-Temporal Graph Attention Network (STGAT) provides interpretable attention patterns across joints and time, yielding compact structural descriptors [8]. However, most methods are developed and evaluated in centralized settings and do not address privacy constraints.

**Figure 1:**
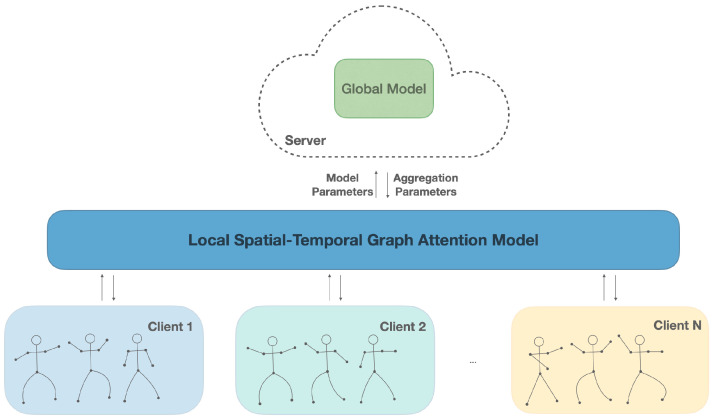
Overview of Fed-STGAT. Each client trains a local STGAT on its private skeleton sequences and periodically uploads model updates. The server aggregates the received updates to obtain the global model and broadcasts it back to clients for the next round. Raw data never leaves the clients, preserving data privacy.

In practice, skeleton data are compartmentalized between devices or institutions, and privacy rules impede centralization. Federated learning (FL) enables collaborative training without sharing raw data, thereby preserving privacy and supporting personalization [9]. A baseline method is established by integrating STGAT with the standard FedAvg algorithm, referred to as Fed-STGAT (see Figure 1). This configuration preserves data privacy by sharing model parameters, thereby keeping the raw skeleton data streams on the client devices. Nevertheless, non-IID heterogeneity which span subjects, viewpoints, and recording setups induces client drift and unstable convergence when updates are naively averaged. In skeleton HAR the effect is exacerbated: the same action may manifest distinct dynamics across subjects and capture setups, inflating variance and degrading global averaging. Existing FL-HAR and clustered FL partially alleviate these issues. Prior efforts with regularized or contrasting objectives (e.g., FedProx [10], MOON [11]) constrain local training, while clustered FL (e.g., FedCLAR [12]) groups similar clients before aggregation to reduce cross-client variance and stabilize optimization. However, few methods explicitly leverage attention statistics a privacy-friendly, structure-aware signals for clustering and coordination. Compared to sample-level features, an attention descriptor is a low-dimensional statistic derived from the trained model that summarizes cross-joint and cross-time importance patterns and aligns naturally with the skeleton graphs.

We therefore propose Clustered Federated Spatio-Temporal Graph Attention Networks (CF-STGAT). Clients train STGAT locally; the server periodically extracts lightweight multi-head attention statistics and performs K-means to obtain several structure-aware, homogeneous clusters. Beyond standard within-cluster aggregation, we compute a global reference by attention–similarity weighting (via cosine similarity between cluster-level attention centroids and a global prototype) and apply a lightweight fusion to the intra-cluster aggregates. This design preserves personalization while mitigating cross-cluster drift and adds only minimal server-side statistical and clustering over-head.

The contributions of this paper are summarized as follows:

- We introduce an attention–descriptor-driven federated clustering paradigm for skeleton HAR, turning spatio-temporal attention into a privacy-friendly client representation for dynamic cluster assignment.
- We propose an attention–similarity-weighted cross-cluster fusion that balances personalization and consistency via a lightweight update, without changing the client-side training pipeline.
- We perform comprehensive experiments on NTU 60/120 with X-Sub/X-Setup, benchmarking against FedAvg, FedProx, MOON, and clustered baselines FedCLAR. CF-STGAT achieves consistent accuracy gains and smoother convergence.

## 2. Related Work

### 2.1. Spatio-Temporal Model for Skeleton-Based Action Recognition

Modeling spatio-temporal dependencies is central to skeleton-based action recognition. Beyond early CNN/TCN pipelines [13,14,15], recent 3D-CNNs designs revisit skeleton encoding with task-specific representations, for example, PoseConv3D [16] encodes poses as 3D heatmap volumes and achieves strong cross-dataset robustness. Graph-based methods continue to advance topology modeling: InfoGCN [17] integrates an information-bottleneck objective with attention-based graph convolution to infer context-dependent topology, HD-GCN hierarchically decomposes skeleton graphs to capture semantically meaningful edges [18], ref. [19] reviewed skeleton graph neural network-based human action recognition, and BlockGCN [20] redefines topology awareness via block-wise graph convolutions with improved efficiency. Transformer-style models further extend receptive fields, FG-STFormer [21] balances focal/local and global attention, STAR-Transformer employs cross-attention to fuse spatio-temporal cues [22], and SkateFormer partitions joints and frames to efficiently model skeletal–temporal relations [23]. While attention improves global dependency modeling, its computational cost can be high, efficient self-attention and local dynamic STGAT reduce over-head while maintaining accuracy [8,24]. Recent surveys synthesize the field’s shift from RNN/LSTM to GCN/GAT/Transformer families and highlight the need for structure-aware, efficient, and privacy-preserving learning, which is the target setting of our framework CF-STGAT [25,26].

### 2.2. Federated Learning for Skeleton-Based Action Recognition

Federated learning (FL) enables privacy-preserving collaboration by keeping raw data on device and exchanging only model updates, thereby reducing both data-exposure risk and the costs of centralized collection; in practice, it is often paired with secure aggregation and lightweight communication protocols, making FedAvg [27] a widely adopted, communication-efficient baseline for deep models across modalities [28,29,30]. To address non-IID data, regularized and contrastive formulations improve robustness to statistical heterogeneity, FedProx [10] augments the local objective with a proximal term to constrain client drift under variable local steps, while MOON [11] introduces a model-contrastive objective that aligns client and global representations to reduce drift. Variance-reduction and normalization strategies such as SCAFFOLD and FedNova further correct client-specific bias and the objective inconsistency induced by unbalanced local updates [31,32], yet these methods remain structure-agnostic to skeleton topology. Furthermore, clustered and personalized schemes address client heterogeneity that is typical in skeleton HAR, where agglomerative clustered FL groups clients by update similarity to stabilize training [12,33], while structured FL via clustered additive modeling provides a principled formulation to reconcile cluster-wise personalization with global coordination [34]; however, they rely on generic similarity measures that do not explicitly reflect spatio-temporal skeleton dependencies. When skeleton streams and their topologies are explicitly modeled as graphs, federated graph learning (FGL) [35] offers task-tailored aggregation and topology-aware updates, recent systems and algorithms demonstrate gains by combining global coordination with local graph-specific objectives [36,37]. Related cross-silo variants such as vertical FL and split learning are also relevant when different parties hold complementary features or wish to offload parts of the network; recent surveys and studies document privacy risks and corresponding defenses, as well as practical split feature-partitioned training pipelines that can be combined with FL for spatio-temporal data [38,39,40]. In contrast, we adopt FedAvg as the baseline privacy boundary and inject structure awareness through leveraging parameter-based attention features from STGAT to drive clustering and attention–similarity-weighted fusion, achieving cluster-wise personalization while gently regularizing cross-cluster drift.

## 3. Methods

### 3.1. Preliminary

We adopt STGAT as the local model due to its strong empirical performance in capturing short-term features for skeleton sequences via spatio-temporal patterns. Below, we formalize the graph construction and summarize the network components relevant to our method.

#### 3.1.1. Spatio-Temporal Graph Building

Formally, the human skeleton graph is always represented as G=(V,A), where $V=\{v_{1},v_{2},\ldots ,v_{N}\}$ denotes skeleton joints and $A\in R^{N\times N}$ is the adjacency matrix. $A_{i,j}\neq 0$ indicates a connection between joint $v_{i}$ and $v_{j}$, otherwise $A_{i,j}=0$ without connections. Regarding the input, the action sequences are characterized by a set of node features, represented as a feature tensor $X\in R^{C\times T\times N}$ where each joint is described by a *C* dimensional feature vector across *T* frames. To enhance the capture of local cross-spacetime joint relationships without necessitating multiple transmissions, the construction of a spatio-temporal graph is proposed in which joints within a localized cross-spacetime neighborhood are represented as nodes. These nodes are connected by edges not only to other nodes within their original spatio graph but also to their counterparts in adjacent temporal frames. For a given time point *t* and window size $\tau \in N^{*}$, the concatenation of features within the time window can be expressed as $X_{\tau }^{t}=[X_{:,t-\frac{\tau }{2},:};X_{:,t-\frac{\tau }{2}+1,:};\ldots ;X_{:,t+\frac{\tau }{2},:}]\in R^{C\times \tau \times N}$. Then, a time-aware adjacency is defined as $\tilde{A_{\tau }^{t}}=\left[\tilde{A_{1}^{t}};\ldots ;\tilde{A_{\tau }^{t}}\right]\in R^{\tau \times N\times N}$.

#### 3.1.2. STGAT Network

As is widely recognized, for a graph *G*, with the node feature matrix *X* serving as the input, the formulation for a new feature matrix *H* derived from a layer within a graph convolutional network (GCN) at each timestep can be described by

(1) $$H=\sigma ({\hat{D}}^{-\frac{1}{2}}\hat{A}{\hat{D}}^{-\frac{1}{2}}XW),$$

The degree matrix $\tilde{D}$ corresponds to $\tilde{A}$ and is a diagonal matrix whose elements represent the sum of the connections, including self-loops, that incorporate its own features into the calculation. Also, *W* typically represents the trainable weight matrix, and σ denotes a non-linear activation function. In parallel to the development of the spatio-temporal graph, by integrating $\tilde{A_{\tau }^{t}}$ into graph operations, the output for each timestep input $X_{\tau }^{t}$ can be derived as

(2) $$H_{\tau }^{t}=\sigma \left(\tilde{A_{\tau }^{t}}X_{\tau }^{t}W\right),$$

Furthermore, STGAT utilizes multi-head attention modules to learn various kinds of edge weights. Specifically, independent self-attention modules *S* are employed to discern diverse graph structures. Consequently, the aggregated output from all heads across various spatio-temporal graphs can be expressed as

(3) $$Y^{t}=\sigma \left(\frac{1}{S}\sum_{s=1}^{S}{\hat{A}}_{\tau }^{t}{,}_{s}X_{\tau }^{t}W_{s}\right),$$

where ${\hat{A}}_{\tau }^{t}{,}_{s}$ denotes the unique spatio-temporal graph computed at timestamp *t*, with a temporal length of τ for the $s_{th}$ head.

### 3.2. Fed-STGAT

As illustrated in Figure 1, the structure comprises N distributed clients and a central aggregation server. Each client trains a local STGAT on private data and uploads model parameters Θ after local updates. The server aggregates the received parameters and broadcasts the global model for the next round, repeating until convergence or a fixed communication budget.

#### 3.2.1. Local Updates

For each client i∈N, at the beginning of each communication round, the local STGAT model is initialized with the global model parameters $\Theta^{t}$ received from the server. The model is then trained on the client’s private dataset using multiple steps of SGD to perform parameter updates. The detailed procedure is as follows:

(4) $$\Theta_{i}^{t+1}=\Theta^{t}-\eta \;\cdot \;\nabla_{\Theta }\;L\;\left(\Theta^{t};\;D_{i}\right).$$

where η is the learning rate and $\nabla_{\Theta }\;L\;\left(\Theta^{t};\;D_{i}\right)$ is the set of gradient updates of the local dataset on client *i*. Upon completion of the local training, each client uploads its updated parameters to the central server for aggregation.

#### 3.2.2. Aggregation

The server performs global model aggregation when receiving the locally updated model parameters ${\left\{\Theta_{i}^{t}\right\}}_{i=1}^{N}$ from N participating clients. The global model is updated on FedAvg as follows:

(5) $$\Theta^{t+1}=\sum_{i=1}^{N}\frac{n_{i}}{n}\Theta_{i}^{t},$$

where $n_{i}$ denotes the number of the local data samples on client *i*, and *n* is the total number of samples from all selected clients. The updated global parameters are redistributed to clients for the next round.

#### 3.2.3. Limitation of Fed-STGAT

Under non-IID partitions, FedAvg can induce client drift and unstable convergence. The lack of structure-aware aggregation and personalization further limits cross-subject generalization. Thus, we introduce dynamic clustering in Section 3.3 and a light fusion step in Section 3.4 to mitigate drift and reduce inter-client variance.

### 3.3. Dynamic Cluster Adjustment

The approach extends FedAvg by grouping clients dynamically into clusters and training cluster-specific models. To obtain a structure-aware similarity, we extract attention-layer parameters from each client’s STGAT as privacy-preserving features that reflect spatio-temporal patterns. Every $I_{c}$ rounds, the server reclusters participating clients based on these features. Before clustering, we normalize the feature vector of each client $a_{i}=\left[a_{i1},\ldots ,a_{im}\right]$ by Z-score, then we fit a PCA model on the normalized attention vectors to reduce the dimensionality from *m* to *d* automatically by retaining 90% of the cumulative explained variance. This yields low-dimensional descriptors that preserve most of the information in the original attention parameters while reducing noise and improving the efficiency and stability of the subsequent clustering step. Having assembled the matrix $\tilde{A}=\left[{\tilde{a}}_{1},\ldots ,{\tilde{a}}_{n}\right]$, the empirical covariance matrix is computed as $\Sigma =\frac{1}{n}\;{\tilde{A}}^{⊤}\tilde{A}$. Solving the eigenproblem $\Sigma U_{d}=U_{d}\Lambda_{d}$ yields the top-deigenvectors $U_{d}=\left[u_{1},\ldots ,u_{d}\right]$ associated with the largest eigenvalues in $\Lambda_{d}$. Each normalized vector ${\tilde{a}}_{i}$ is then projected as

(6) $$z_{i}=U_{d}^{⊤}{\tilde{a}}_{i},$$

yielding the final *d*-dimensional clustering feature for client *i*. If the current round is one of the designated clustering intervals $I_{c}$, the server uses the collected feature vectors from all participating clients to recompute the client clusters. If a client is assigned to another cluster, it means the characteristics of its model have shifted or the clustering decision boundary has changed.

**Figure 2:**
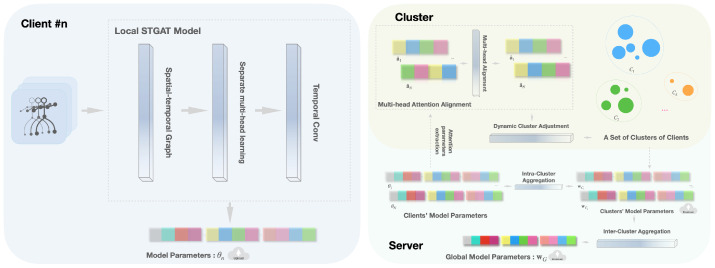
CF-STGAT pipeline. (**Left**) Each client trains a local STGAT and uploads model parameters $\theta_{n}$ after *E* local steps. (**Right, top**) The server extracts and normalizes attention statistics and performs multi-head attention alignment; every $I_{c}$ rounds, a dynamic clustering partitions clients into $\left\{C_{1},\ldots ,C_{C}\right\}$. (**Right, bottom**) Inter-cluster aggregation forms an attention-weighted global reference $w_{G}$ and updates each cluster model by $w_{C_{k}}\leftarrow \left(1-\gamma \right)w_{C_{k}}+\gamma \;w_{G}$ before the next round. Notation: γ∈[0,1] fusion coefficient; *E* local steps; $I_{c}$ reclustering interval.

We adopt a K-means approach for the clustering algorithm due to its simplicity and efficiency in the federated setting, partitioning the clients into *C* clusters by minimizing the within-cluster variance in the feature space. As shown in Figure 2, the outcome is a set of clusters $C_{1},C_{2},\ldots ,C_{C}$, where clients within each cluster have high internal similarity in their attention features $z_{i}$. After clustering, each cluster $C_{k}$ is associated with its own sub-model. Clients do not remain tied to a single cluster for the entire training; they are allowed to change clusters if their attention-based representation shifts or the clustering boundaries are refined over time.

Complexity note. Let *N* denote the number of participating clients in a clustering step and *m* the dimensionality of the raw attention features. Computing the covariance matrix and its eigendecomposition for PCA on the N×m matrix $\tilde{A}$ has complexity $O(Nm^{2}+m^{3})$. Since *m* is a small, fixed hyperparameter in our setup, this cost is effectively linear in *N*. Performing K-means in the reduced *d*-dimensional space has complexity O(NCId), where *C* is the number of clusters and *I* is the number of iterations. In practice, *m*, *d*, *C*, and *I* are all small constants, and clustering is only performed every $I_{c}$ rounds on the subset of active clients, so the server-side over-head is negligible compared to local STGAT training and communication.

### 3.4. Cluster Federated Aggregation

While clustered federated learning provides personalization by clustering models to groups of similar clients, it is important to prevent these cluster-specific models from diverging too far from each other, which could harm overall generalization. Therefore, for Cluster $C_{k}$, the server forms a size-weighted average ${\bar{w}}_{C_{k}}$ of the received client models; for inter-cluster regularization aggregation, to maintain global coherence between clusters and prevent cluster models from diverging excessively.

We construct a global attention prototype $a_{G}$ from cluster attention centroids and compute cosine similarities $s_{k}=cosine\left(a_{k},a_{G}\right)$ between cluster $C_{k}$. Attention-aware weights

(7) $$\alpha_{k}=\frac{N_{k}\;s_{k}}{\sum_{j}N_{j}\;s_{j}},\;\sum_{k}\alpha_{k}=1.$$

which yield a reference model:

(8) $$w_{G}^{\left(t\right)}=\sum_{k}\alpha_{k}{\bar{w}}_{C_{k}}^{\left(t\right)}.$$

Each cluster model is then updated by a light fusion:

(9) $${\bar{w}}_{C_{k}}^{\left(t+1\right)}\leftarrow \left(1-\gamma \right)\;{\bar{w}}_{C_{k}}^{\left(t\right)}+\gamma \;w_{G}^{\left(t\right)},$$

with γ controlling inter-cluster regularization. Instead of treating the fusion coefficient γ as a fixed hyperparameter, we adapt it automatically at each communication round based on the discrepancy between the previous global model and the current cluster models. In practice, this makes γ increase when cluster models deviate more from the global model (encouraging stronger fusion) and decrease when the models are already similar (allowing more specialization). This step preserves local training pipelines while aligning clusters through periodic server-side coordination to effectively balance model personalization with overall consistency in a federated learning setting.

The above formulation can be understood as follows. The server clusters clients through similar attention-based descriptors which form the uploaded spatio-temporal structure, so that each cluster-wise model is trained on a group of clients that share similar dependency patterns. The fusion regularization term softly couples these cluster-specific models, allowing them to share transferable knowledge while still adapting to the particular characteristics of each cluster. In this way, the method balances global sharing and local specialization in a structure-aware manner.

## 4. Experimental Results

### 4.1. Datasets

NTU 60 [41]. A widely adopted benchmark for 3D skeleton-based human action recognition. Captured with Microsoft Kinect v2 sensors in a controlled laboratory setting, it comprises 56,880 samples across 60 action classes performed by 40 subjects. Each sample encodes a sequence of 25 skeletal joints, with each joint represented by its (x, y, z) coordinates in 3D space. The dataset offers two standardized evaluation protocols: cross-subject (X-Sub), which trains on half of the subjects and tests on the other half, and cross-view (X-View). We follow the standard X-Sub split; for X-View, we instead use a setup-based split for stress-testing distribution shift.

NTU 120 [42]. An extended version of NTU 60 that includes 120 action classes, each performed twice by 60 subjects (40 male, 20 female) across four distinct recording setups. NTU 120 retains the same data modalities as NTU 60, offering synchronized RGB and depth streams along with the core 3D skeleton data (25 joints per frame). NTU 120 introduces the Cross-Setup (X-Setup) protocol: even-ID setups for training and odd-ID setups for testing. Each setup features a distinct location and background and, as in NTU 60, it employs three horizontally spaced cameras to capture each action from three different side-view angles simultaneously.

We also adopt a federated-by-dataset setting, where federated clients are constructed from the underlying dataset structure. Concretely, for both NTU 60 and NTU 120, each (subject,setup) pair thus defines a federated client, inducing a naturally non-IID label and setup distribution across clients. The statistics of federated data partitions are shown in Table 1. The central server evaluates on a global test set defined over subject–setup pairs. For NTU 60, in addition to the standard X-Sub, we define a cross-setup protocol (NTU 60 X-Setup) to stress-test distribution shifts across recording setups: following the rule of NTU 120 X-Setup, sequences from even-numbered setups are used for training and those from odd-numbered setups for testing.

### 4.2. The Details of Implementation

All experiments are conducted using the PyTorch 1.12.1 on a single NVIDIA RTX 4090 GPU. The backbone network is based on STGAT, consisting of eight graph attention layers, each with eight attention heads. We use batch size 32 and weight decay $5\times 10^{-4}$. From each sequence, we first sample 150 frames and then randomly crop to 128 frames for both training and testing to match sequence length. Within FL, each client performs E=1 local epoch per communication cycle. The initial learning rate is set to 0.1 and is reduced by a factor of 10 at communication rounds {60,90,120}, respectively. The total number of communication rounds is fixed at R=200. For clustering, the interval is $I_{c}=5$ communication rounds. We set C=4 on NTU 60 and C=6 on NTU 120. The fusion regularization coefficient γ is selected from the set {0.1,0.2,0.5} based on a small grid search.

### 4.3. Experiments Results and Discussion

#### 4.3.1. General Results

Table 2 presents a comparative performance analysis on NTU 60/120 under the X-Sub and X-Setup protocols with STGAT as the backbone. (1) CF-STGAT achieves the best overall performance, surpassing FedAvg by +0.84 (NTU 60, X-Sub), +4.09 (NTU 60, X-Setup), +7.98 (NTU 120, X-Sub), and +4.18 (NTU 120, X-Setup), yielding an average gain of +4.27 percentage points (pp). The largest single-cell improvement occurs on NTU 120 (X-Sub) (+7.98). (2) CF-STGAT also outperforms FedCLAR across all settings, with gains of +0.68, +3.31, +4.78, and +2.06 pp on the same four protocol–dataset combinations (mean +2.71 pp). Notably, the margin is largest on NTU 120 (X-Sub) (+4.78). (3) CF-STGAT Improvements over FedAvg are larger on NTU 120 (mean +6.08 pp) than on NTU 60 (mean +2.47 pp), suggesting greater benefits under the more heterogeneous setting. Overall, relative to optimizer-based baselines (FedAvg, FedProx, MOON) and clustered FL (FedCLAR), CF-STGAT attains the highest accuracy across all protocols, with robust gains on NTU 120.

#### 4.3.2. Clustering Stability Analysis

To analyze how client cluster assignments evolve over communication rounds, we measure round-to-round agreement between consecutive reclustering checkpoints using the Adjusted Rand Index (ARI) and report a churn rate (the fraction of clients that change clusters between two checkpoints). Across NTU 60 X-Sub configurations, the reclustering dynamics stabilize after a short warm-up. As shown in Table 3, the $I_{c}=5$, C=4 variant yields the strongest consistency (ARI = 0.846 ± 0.213) and the lowest churn (0.040 ± 0.056), outperforming C=3 (ARI = 0.674 ± 0.326; churn = 0.064 ± 0.077) and $I_{c}=3$ (ARI = 0.750 ± 0.236; churn = 0.064 ± 0.064). This indicates that too small *C* conflates heterogeneous clients, producing fuzzy boundaries. Conversely, when *C* is too large, semantically similar clients are over-partitioned, leading to cluster fragmentation and elevated churn. Also, overly frequent reclustering (smaller $I_{c}$) can introduce transient boundary oscillations, even if the fraction of migrating clients is moderate, the induced pairwise disagreements depress ARI.

**Figure 3:**
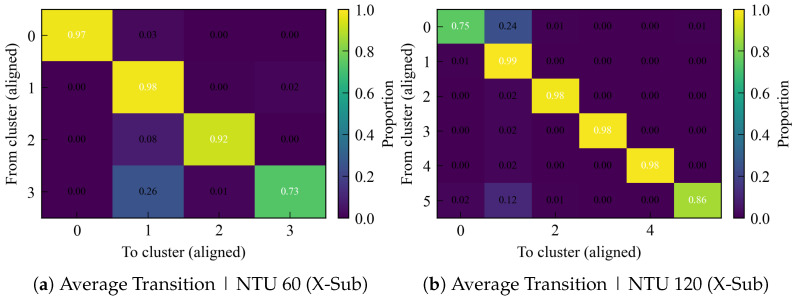
Row-normalized average transition matrices across consecutive checkpoints. Values are averaged over all checkpoint pairs after Hungarian label alignment.

In addition, we visualize the row-normalized average transition matrix to reveal where changes concentrate. The transition matrices in Figure 3 are strongly diagonal on both datasets, confirming persistent cluster identities. On NTU 120, clusters 1–4 exhibit near-perfect self-transition (≥0.98), with localized drift from 0→1 (0.24) and 5→1 (0.12), that means a single fuzzy boundary while the rest remain stable. On NTU 60, overall stability is also high, but the boundary between clusters shows more activity: 3→1≈0.26 and 2→1≈0.08, consistent with the slightly lower ARI mean and the small but non-zero churn pulses. Overall, the table and heatmaps show that instability is concentrated at a few adjacent-cluster boundaries rather than reflecting global oscillations.

#### 4.3.3. Effectiveness of Clustering

**Figure 4:**
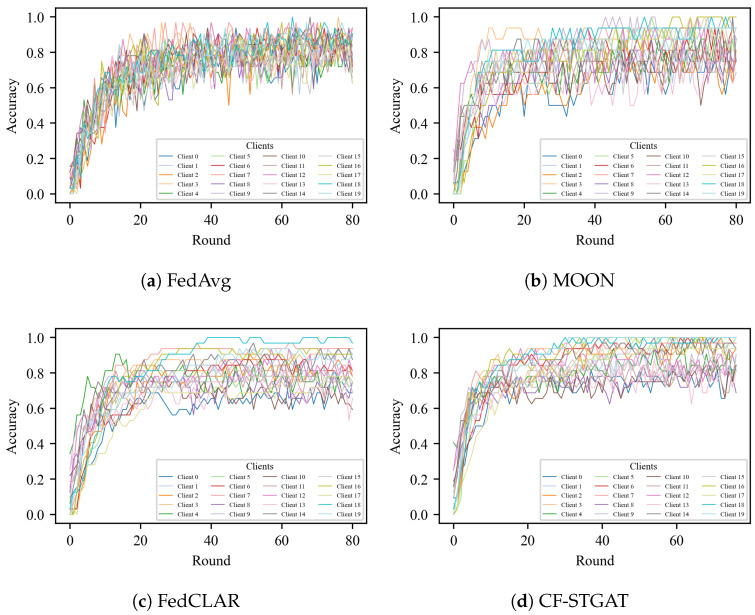
Per-client training accuracy vs. communication rounds on NTU RGB+D 60 (X-Sub). (**a**) FedAvg. (**b**) MOON. (**c**) FedCLAR (clustered-only). (**d**) CF-STGAT (clustering + global fusion). Each curve denotes individual clients; tighter bundles indicate lower dispersion.

**Figure 5:**
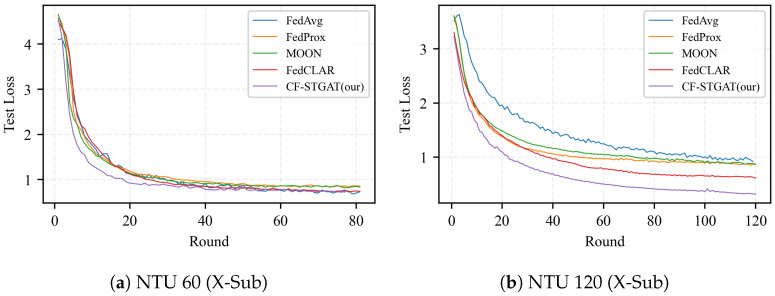
Test loss vs. communication rounds. (**a**) NTU 60 (X-Sub): CF-STGAT(ours) remains lowest throughout. (**b**) NTU 120 (X-Sub): separation is pronounced. CF-STGAT(ours) attains the fastest decay and lowest asymptote (lower is better).

As shown in Figure 4, in the absence of clustering (treating all clients as a single group), the baseline method FedAvg and its regularized extensions FedProx and MOON exhibit slow and uneven convergence under non-IID partitions. Training a single global model with FedAvg on highly non-IID clients can induce large variance in the aggregated update, since local gradients are optimized for very different data distributions. This variance manifests as oscillatory behavior of the global model and unstable performance across communication rounds. Although MOON accelerates the initial rise, the jagged behavior remains, indicating that optimizer-level regularization alone is insufficient to eliminate client drift. Consistently, the test loss shown in Figure 5 decreases more slowly and stabilizes at a higher asymptote, particularly on NTU 120, where label and domain shifts are stronger. By contrast, our clustering mechanism groups clients with similar attention-based descriptors, i.e., similar spatio-temporal dependency patterns over the skeleton graph, so that each cluster-wise model is trained on a more homogeneous subset of clients. Within a cluster, local updates are better aligned and the variance of the aggregated update is reduced, which mitigates client drift and yields smoother training trajectories. The resulting cluster-specific models thus exhibit more stable convergence behavior than a single global model trained on all heterogeneous clients.

#### 4.3.4. Effectiveness of Fusion

With the clustered-only method FedCLAR, most clients indeed benefit from more homogeneous aggregation; however, the persistent low-accuracy tail and residual jaggedness indicate that inter-cluster drift is not fully controlled. Compared with our approach, this limitation is reflected in a higher late-stage test loss (Figure 5). By contrast, CF-STGAT, clustering augmented with global fusion, produces tighter and smoother client trajectories, elevates the plateau height, and compresses the long tail (Figure 4). On the test set, fusion yields faster loss decay and a lower terminal loss, with the largest margin observed on NTU 120. Overall, these results show that our method complements clustering by gently yet effectively aligning clusters, thereby delivering additional accuracy and stability beyond clustered-only federated learning.

#### 4.3.5. Discussion and Future Work

A limitation of the present study is that we do not include explicit empirical comparisons with some recent personalized federated learning methods, such as FedPer [43] and FedRep [44]. These approaches adopt a complementary perspective: they typically share a global feature extractor while learning client-specific heads or representations, thereby personalizing predictions at the client level without explicitly modeling relationships among clients. In contrast, our method focuses on structure-aware clustering of clients based on attention-derived descriptors and trains cluster-specific STGAT models to capture shared spatio-temporal patterns within each group. This design is orthogonal to head- or representation-based personalization and could, in principle, be combined with FedPer/FedRep-style techniques by applying them within each discovered cluster. We leave a systematic empirical comparison and such hybrid combinations as important directions for future work. From a privacy perspective, the server only observes compressed attention-based descriptors aggregated over each client, rather than raw data or per-sample activations; while this reduces the risk of direct data leakage, our method is complementary to secure aggregation and differential-privacy mechanisms, and a formal privacy analysis of such descriptors is left for future work.

## 5. Conclusions

In summary, this work introduced CF-STGAT, a clustered federated framework for skeleton-based action recognition that integrates multi-head spatio-temporal attention with dynamic client clustering and a lightweight, attention-weighted inter-cluster fusion. The approach preserves cluster-level personalization while providing gentle cross-cluster alignment. Empirically, CF-STGAT delivers higher accuracy, smoother and faster convergence, and consistently lower terminal test loss, with the advantages becoming more pronounced under stronger heterogeneity. The method leaves client-side training unchanged and adds only modest server-side statistics and clustering.

## Figures and Tables

**Table 1:** Statistics of the federated data partitions on NTU 60 and NTU 120.

Dataset	Protocol	Classes	Train Clients	Test Clients
NTU 60	X-Sub	60	20	20
NTU 60	X-Setup	60	8	9
NTU 120	X-Sub	120	53	53
NTU 120	X-Setup	120	16	16

**Table 2:** Overall comparison on NTU RGB+D 60/120 under X-Sub and X-Setup. We report top-1 accuracy (%) on each protocol. Bold denotes the best. Values in parentheses indicate the absolute margin vs. FedAvg (baseline row marked with *).

Model Backbone: STGAT	NTU 60		NTU 120	
	X-Sub (%)	X-Setup (%)	X-Sub (%)	X-Setup (%)
Fed-STGAT *	89.72	86.46	72.35	78.71
FedProx	89.31 (−0.41)	87.49 (+1.03)	75.36 (+3.01)	80.65 (+1.94)
MOON	88.01 (−1.71)	86.96 (+0.50)	73.31 (1.04)	79.86 (+1.15)
FedCLAR	89.88 (+0.16)	87.24 (+0.78)	75.55 (+3.20)	80.83 (+2.12)
CF-STGAT (our)	**90.56** (+0.84)	**90.55** (+4.09)	**80.33** (+7.98)	**82.89** (+4.18)

**Table 3:** Round-to-round clustering stability on NUT 60 (X-Sub). Values are mean ± std over consecutive checkpoint pairs.

Setup	ARI	Churn
$I_{c}=5$, C=4	0.846 ± 0.213	0.040 ± 0.056
$I_{c}=5$, C=3	0.674 ± 0.326	0.064 ± 0.077
$I_{c}=3$, C=4	0.750 ± 0.236	0.064 ± 0.064

## Data Availability

The data presented in this study are available in [NTU RGB+D] at [https://doi.org/10.1109/CVPR.2016.115], reference number [41].

## References

[1] Dai C., Lu S., Liu C., Guo B. (2024). A light-weight skeleton human action recognition model with knowledge distillation for edge intelligent surveillance applications. Appl. Soft Comput..

[2] Terreran M., Barcellona L., Ghidoni S. (2023). A general skeleton-based action and gesture recognition framework for human–robot collaboration. Robot. Auton. Syst..

[3] Yan S., Xiong Y., Lin D. Spatial temporal graph convolutional networks for skeleton-based action recognition. Proceedings of the AAAI Conference on Artificial Intelligence.

[4] Xiao L., Yang X., Peng T., Li H., Guo R. (2024). Skeleton-Based Activity Recognition for Process-Based Quality Control of Concealed Work via Spatial–Temporal Graph Convolutional Networks. Sensors.

[5] Shi L., Zhang Y., Cheng J., Lu H. Two-stream adaptive graph convolutional networks for skeleton-based action recognition. Proceedings of the IEEE/CVF Conference on Computer Vision and Pattern Recognition.

[6] Chen Y., Zhang Z., Yuan C., Li B., Deng Y., Hu W. Channel-wise topology refinement graph convolution for skeleton-based action recognition. Proceedings of the IEEE/CVF International Conference on Computer Vision.

[7] Liu Z., Zhang H., Chen Z., Wang Z., Ouyang W. Disentangling and unifying graph convolutions for skeleton-based action recognition. Proceedings of the IEEE/CVF Conference on Computer Vision and Pattern Recognition.

[8] Hu L., Liu S., Feng W. (2023). Skeleton-based action recognition with local dynamic spatial–temporal aggregation. Expert Syst. Appl..

[9] Li C., Niu D., Jiang B., Zuo X., Yang J. Meta-har: Federated representation learning for human activity recognition. Proceedings of the Web Conference 2021.

[10] Li T., Sahu A.K., Zaheer M., Sanjabi M., Talwalkar A., Smith V. Federated optimization in heterogeneous networks. Proceedings of the Machine Learning and Systems.

[11] Li Q., He B., Song D. Model-contrastive federated learning. Proceedings of the IEEE/CVF Conference on Computer Vision and Pattern Recognition.

[12] Presotto R., Civitarese G., Bettini C. (2022). Fedclar: Federated clustering for personalized sensor-based human activity recognition. Proceedings of the 2022 IEEE International Conference on Pervasive Computing and Communications (PerCom).

[13] Kim T.S., Reiter A. (2017). Interpretable 3d human action analysis with temporal convolutional networks. Proceedings of the 2017 IEEE Conference on Computer Vision and Pattern Recognition Workshops (CVPRW).

[14] Lea C., Flynn M.D., Vidal R., Reiter A., Hager G.D. Temporal convolutional networks for action segmentation and detection. Proceedings of the IEEE Conference on Computer Vision and Pattern Recognition.

[15] Nguyen H.C., Nguyen T.H., Scherer R., Le V.H. (2023). Deep learning for human activity recognition on 3D human skeleton: Survey and comparative study. Sensors.

[16] Duan H., Zhao Y., Chen K., Lin D., Dai B. Revisiting skeleton-based action recognition. Proceedings of the IEEE/CVF Conference on Computer Vision and Pattern Recognition.

[17] Chi H.g., Ha M.H., Chi S., Lee S.W., Huang Q., Ramani K. Infogcn: Representation learning for human skeleton-based action recognition. Proceedings of the IEEE/CVF Conference on Computer Vision and Pattern Recognition.

[18] Lee J., Lee M., Lee D., Lee S. Hierarchically decomposed graph convolutional networks for skeleton-based action recognition. Proceedings of the IEEE/CVF International Conference on Computer Vision.

[19] Feng M., Meunier J. (2022). Skeleton graph-neural-network-based human action recognition: A survey. Sensors.

[20] Zhou Y., Yan X., Cheng Z.Q., Yan Y., Dai Q., Hua X.S. Blockgcn: Redefine topology awareness for skeleton-based action recognition. Proceedings of the IEEE/CVF Conference on Computer Vision and Pattern Recognition.

[21] Gao Z., Wang P., Lv P., Jiang X., Liu Q., Wang P., Xu M., Li W. Focal and global spatial-temporal transformer for skeleton-based action recognition. Proceedings of the Asian Conference on Computer Vision.

[22] Ahn D., Kim S., Hong H., Ko B.C. Star-transformer: A spatio-temporal cross attention transformer for human action recognition. Proceedings of the IEEE/CVF Winter Conference on Applications of Computer Vision.

[23] Do J., Kim M. (2024). Skateformer: Skeletal-temporal transformer for human action recognition. Proceedings of the European Conference on Computer Vision.

[24] Qin X., Cai R., Yu J., He C., Zhang X. (2022). An efficient self-attention network for skeleton-based action recognition. Sci. Rep..

[25] Xin W., Liu R., Liu Y., Chen Y., Yu W., Miao Q. (2023). Transformer for skeleton-based action recognition: A review of recent advances. Neurocomputing.

[26] Ren B., Liu M., Ding R., Liu H. (2024). A survey on 3d skeleton-based action recognition using learning method. Cyborg Bionic Syst..

[27] McMahan B., Moore E., Ramage D., Hampson S., y Arcas B.A. Communication-efficient learning of deep networks from decentralized data. Proceedings of the Artificial Intelligence and Statistics, PMLR.

[28] Li Q., Wen Z., Wu Z., Hu S., Wang N., Li Y., Liu X., He B. (2021). A survey on federated learning systems: Vision, hype and reality for data privacy and protection. IEEE Trans. Knowl. Data Eng..

[29] Liu B., Lv N., Guo Y., Li Y. (2024). Recent advances on federated learning: A systematic survey. Neurocomputing.

[30] Zhao J., Bagchi S., Avestimehr S., Chan K., Chaterji S., Dimitriadis D., Li J., Li N., Nourian A., Roth H. (2025). The federation strikes back: A survey of federated learning privacy attacks, defenses, applications, and policy landscape. ACM Comput. Surv..

[31] Karimireddy S.P., Kale S., Mohri M., Reddi S., Stich S., Suresh A.T. Scaffold: Stochastic controlled averaging for federated learning. Proceedings of the International Conference on Machine Learning, PMLR.

[32] Wang J., Liu Q., Liang H., Joshi G., Poor H.V. Tackling the objective inconsistency problem in heterogeneous federated optimization. Proceedings of the 34th International Conference on Neural Information Processing Systems, Vancouver BC Canada.

[33] Mehta M., Shao C. (2023). A greedy agglomerative framework for clustered federated learning. IEEE Trans. Ind. Informatics.

[34] Ma J., Zhou T., Long G., Jiang J., Zhang C. Structured federated learning through clustered additive modeling. Proceedings of the 37th International Conference on Neural Information Processing Systems.

[35] Chen C., Xu Z., Hu W., Zheng Z., Zhang J. (2024). FedGL: Federated graph learning framework with global self-supervision. Inf. Sci..

[36] Li X., Wu Z., Zhang W., Zhu Y., Li R.H., Wang G. (2023). FedGTA: Topology-Aware Averaging for Federated Graph Learning. VLDB Endow..

[37] Huang W., Wan G., Ye M., Du B. Federated graph semantic and structural learning. Proceedings of the Thirty-Second International Joint Conference on Artificial Intelligence.

[38] Liu Y., Lou Y., Liu Y., Cao Y., Wang H. Label leakage in vertical federated learning: A survey. Proceedings of the IJCAI.

[39] Li Z., Yan C., Zhang X., Gharibi G., Yin Z., Jiang X., Malin B.A. (2024). Split learning for distributed collaborative training of deep learning models in health informatics. AMIA Annu. Symp. Proc..

[40] Ye M., Shen W., Du B., Snezhko E., Kovalev V., Yuen P.C. (2025). Vertical federated learning for effectiveness, security, applicability: A survey. ACM Comput. Surv..

[41] Shahroudy A., Liu J., Ng T.T., Wang G. NTU RGB+D: A large scale dataset for 3D human activity analysis. Proceedings of the IEEE Conference on Computer Vision and Pattern Recognition.

[42] Liu J., Shahroudy A., Perez M., Wang G., Duan L.Y., Kot A.C. (2020). NTU RGB+D 120: A large-scale benchmark for 3D human activity understanding. IEEE Trans. Pattern Anal. Mach. Intell..

[43] Arivazhagan M.G., Aggarwal V., Singh A.K., Choudhary S. (2019). Federated learning with personalization layers. arXiv Prepr..

[44] Collins L., Hassani H., Mokhtari A., Shakkottai S. Exploiting shared representations for personalized federated learning. Proceedings of the International Conference on Machine Learning, PMLR.

